# Synchronous Orbital and Gastrointestinal Metastases from Breast Cancer: A Case Report and Review of Literature

**DOI:** 10.1155/2015/282790

**Published:** 2015-05-13

**Authors:** Ramawad Soobrah, Fiona Tsang, Veronica Grassi, Hassan Hirji, Sreelakshmi Mallappa, Robert Reichert

**Affiliations:** ^1^Victoria Hospital, Ministry of Health & Quality of Life, Mauritius; ^2^Ealing Hospital, Uxbridge Road, Southall, Middlesex UB1 3HW, UK; ^3^King's College Hospital, Denmark Hill, London SE5 9RS, UK; ^4^Northwick Park Hospital, Watford Road, Harrow HA13UJ, UK

## Abstract

Breast cancer is the most common malignancy among women and is a significant cause of morbidity and mortality worldwide. With the advent of improved imaging techniques and screening programmes, only a small proportion of women present with metastatic disease. Metastases involving the gastrointestinal (GI) tract and orbit are rare occurrences. We describe the case of a woman with simultaneous GI and orbital metastases from breast cancer who initially presented with abdominal pain and blurred vision and also summarise a review of the literature.

## 1. Introduction

With 5.2 million cases diagnosed in 2008, breast cancer remains the most prevalent neoplasm in women after nonmelanoma skin cancer [1] and is the second leading cause of cancer deaths in women [2]. Histologically, there are two main subtypes of breast cancer: invasive ductal carcinoma (IDC) and invasive lobular carcinoma (ILC). Invasive lobular carcinoma comprises approximately 10% to 15% of these cases and it is reported that its incidence has been increasing over the last several decades, especially in postmenopausal women [3–5]. ILCs have a distinctive growth pattern which can sometimes lead to vague findings on clinical breast examination making it difficult to diagnose; they fail to form palpable discrete nodules like IDC tumours [6]. Lobular carcinomas often do not have characteristic mammographic appearances and present with subtle abnormalities such as focal asymmetry or architectural distortion [7–10]. About 16% of cases are mammographically occult and the reported sensitivity of mammography to detect ILCs varies between 57% and 81% [11]. This makes early clinical and radiological detection of these tumours very challenging, particularly in women with dense breast tissue. Due to such limitations, other modalities such as ultrasonography and magnetic resonance imaging (MRI) are recommended to further evaluate known cases of ILC [12]. About 10% of breast cancers have already metastasized at the time of presentation and the metastatic patterns of IDC and ILC vary considerably [13]. Various studies have reported that ductal carcinomas tend to spread to the lungs, bones, and liver, whereas lobular carcinomas have a tendency to involve the GI tract, gynaecological organs, and the peritoneum/retroperitoneum [13–15]. Gastrointestinal metastases from ILC are rare and many large series have reported an incidence of less than 1% [13, 16, 17].

The first case of orbital metastasis from lung cancer was described by Horner in 1864 [18]. Around 2.5% to 13% of all orbital tumours are metastatic in nature [19]. Orbital tumours from metastatic breast cancer (MBC) are relatively uncommon. In a large observational case series involving 1264 patients, only 4% were found to be metastatic lesions from breast cancer [20]. However, this figure is likely to be an underestimate because smaller asymptomatic lesions are often undiagnosed [21]. Although orbital and GI metastases from lobular carcinomas are unusual, it is important to recognise these entities to enable physicians to make prompt and accurate diagnosis and provide appropriate treatment. To our knowledge this is the only reported case of breast cancer with synchronous metastasis to the orbit and appendix.

## 2. Case Presentation

A 42-year-old lady presented to the emergency department with a three-day history of nausea, vomiting, fever, abdominal pain, and disturbed vision. She also complained of a three-week history of bilateral breast swelling and right-sided breast pain. On examination, she was apyrexial and had a pulse rate of 76 beats per minute. Both breasts were dense with no discrete palpable lumps; however, she was also noted to have enlarged left axillary lymph nodes. Her abdomen was very tender in the right lower quadrant. White cell count and C-reactive protein were within normal limits.

**Figure 1:**
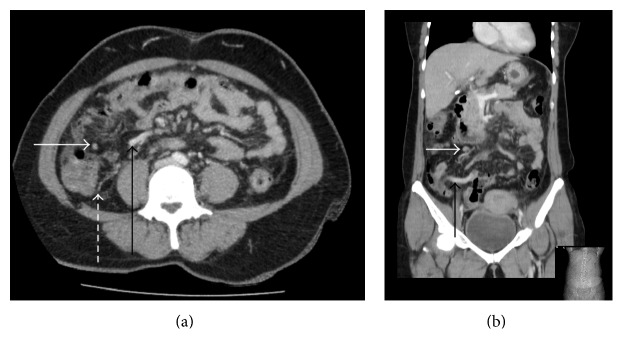
(a) Axial CT with intravenous contrast. The appendix is seen anterior to the right psoas muscle belly and is with a little surrounding free fluid (solid black arrow). Increased attenuating soft tissue in the right paracolic gutter suggests free fluid or secondary peritoneal metastases (broken arrow). (b) Coronal section of the same patient demonstrating the enhancing appendix medial to the caecum. Mural enhancement is commonly seen in appendicitis. However, in this case, there is little surrounding fat stranding and no fluid filled lumen is seen; these features are more atypical. Enlarged lymph nodes are seen in the mesentery (solid white arrow).

**Figure 2:**
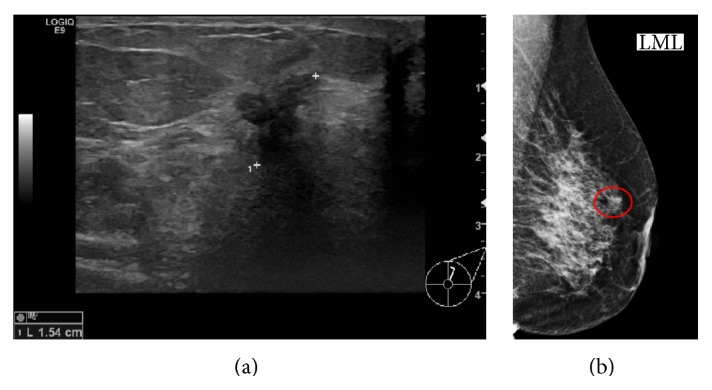
(a) Ultrasound of the left breast showing a 15 mm ill-defined hypoechoic mass. (b) Mammogram of left breast (mediolateral view) demonstrates a spiculate mass (red circle).

A contrast CT scan of the abdomen and pelvis found increased attenuation with mild thickening of the appendix and a small amount of free fluid in the right paracolic gutter; an enlarged lymph node was also seen in the right iliac fossa (Figure 1). A clinical diagnosis of appendicitis was made and the patient underwent an open appendicectomy. During the procedure, a morphologically inflamed appendix was found with abnormal thickening at the base; adjacent small bowel loops were also found to be adherent to the base. The postoperative recovery was uneventful and the patient was discharged home three days later with a planned outpatient breast clinic follow-up. Imaging of the left breast (mammograms and ultrasound scan) showed an ill-defined spiculate mass measuring 30 mm by 27 mm (Figure 2) and enlarged left axillary lymph nodes. Core biopsies of the breast mass and fine needle aspiration (FNA) cytology of the pathological nodes were performed.

**Figure 3:**
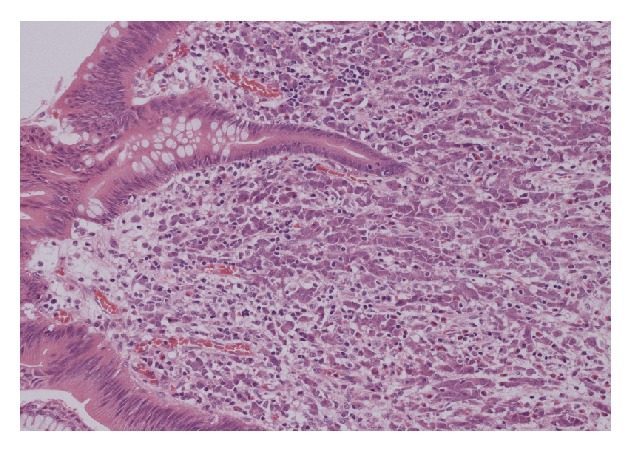
Submucosa shows diffuse infiltration by small to moderate sized malignant epithelial cells with prominent trabecular/Indian filing pattern and vesicular nucleolated nuclei. There is no evidence of glandular formation (H&E stain, magnification ×10).

**Figure 4:**
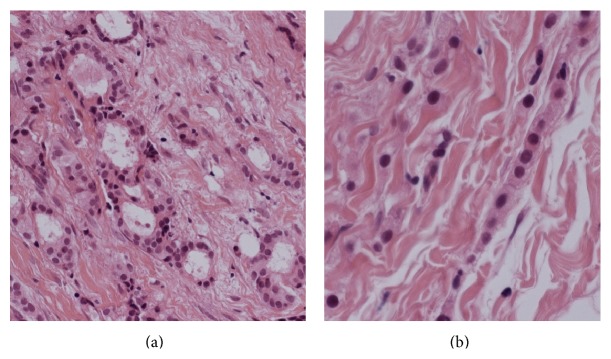
(a) Several malignant ductal structures in a desmoplastic stroma (H&E stain, magnification ×20). (b) Breast biopsy shows typical lobular carcinoma pattern with Indian filing (H&E stain, magnification ×40).

Histology of the appendix showed extensive infiltration of the wall by a tumour which was present in sheets of cells sometimes showing an “Indian filing” pattern. The tumour infiltration also involved the lumen, mesoappendix, and peritoneum. Areas of angiolymphatic invasion were also present (Figure 3). These features were consistent with a metastatic adenocarcinoma of breast origin showing lobular differentiation. The breast core biopsies showed a grade 1 invasive ductal carcinoma with some lobular differentiation (Figure 4) and low grade ductal carcinoma in situ. The FNA of the left axillary lymph node confirmed the presence of metastatic carcinoma. Both tumours were CK7 positive, CK20 and CDX2 negative (the latter precluding a gastrointestinal origin). The tumours were also positive for GCDFP15 (which is consistent with a breast primary), ER positive (7/8), PR positive (8/8), and HER2 negative. Based on the immunohistochemistry results, a diagnosis of metastatic breast cancer was made.

**Figure 5:**
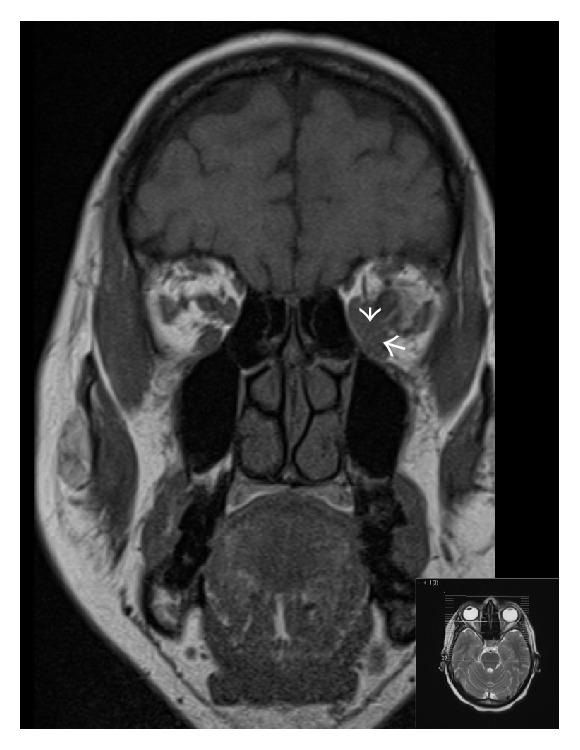
T1 weighted MRI orbits show soft tissue replacing fat in the medial left orbit is isointense with the adjacent recti and it is suggestive of a soft tissue deposit (white arrows).

During the postoperative recovery period the patient noted a worsening of diplopia and left-sided headache. A head CT and contrast-enhanced MRI of the orbits were therefore performed. The MRI scan showed a left retroorbital, intraconal soft tissue mass between the medial and inferior recti muscles, suggestive of a metastatic deposit (Figure 5: MRI orbits). No other significant brain lesions were noted.

The patient completed 6 cycles of chemotherapy (FEC: fluorouracil, epirubicin, and cyclophosphamide), each at 3-weekly interval, and was then started on tamoxifen 20 mg daily. Staging scans (CT and MRI) performed after the chemotherapy and endocrine therapy showed a partial response in all the tumour sites, with reduction of the size of the left breast mass, the axillary, peritoneal, and mesenteric nodes. The orbital metastasis had also reduced in size and the patient noticed an improvement in her vision. Repeat whole body staging CT scan 9 months later showed further marginal reduction of the previously noted left orbital mass and no overt intra-abdominal pathology. Given the noticeable improvement in her condition, she currently remains under close clinical follow-up with the oncology team.

## 3. Discussion

According to 2012 GLOBOCAN estimates, there were nearly 1.7 million new cases of breast cancer diagnosed worldwide [22] and this figure is expected to rise to 2.1 million by 2030 [1]. Extrahepatic gastrointestinal metastases are rare with the stomach reported as the most common site involved, followed by the small intestine and colon [3, 17, 23]. Several large studies have reported that less than 1% of patients with metastatic breast cancer have GI involvement [13, 16, 17]. On autopsy studies, however, this incidence varies between 8% and 35% [24–26]. This discrepancy can be explained by the fact that a large number of cases may be undiagnosed because of their nonspecific symptoms and late presentation. The most common presenting symptoms are abdominal pain followed by bloating, melena, GI hemorrhage, bowel obstruction, nausea and vomiting, early satiety, dysphagia, weight loss, anaemia or fatigue, and palpable mass [13]. Patients present with gastrointestinal metastases after an average of 7 years [13] and as long as 30 years from initial diagnosis of breast cancer [27]. Moreover, GI metastases can present as the first manifestation in clinically occult primary breast cancer [23]. In a large retrospective study, Iorfida et al. showed that independent risk factors for metastatic disease included positive axillary node status, tumours over 2 cm in size, and positive HER2 status [28]. Interestingly, our patient presented with two of these features.

Breast cancer metastasizing to the appendix is also a rare occurrence [23] and there are very few published reports of MBC presenting as appendicitis [29–31]. McLemore et al. [13] reported that 21% of patients presented with metastatic disease masquerading as an alternate disease process, thus making accurate diagnosis difficult. In our case, the patient presented initially with GI symptoms suggestive of possible appendicitis which made her seek medical advice, whilst disregarding her breast complaints. Breast carcinoma has the potential for widespread dissemination and virtually any organ can be involved. Among women, it is the most common primary malignancy to metastasize to the GI tract and is second only to melanoma [24, 32]. The metastatic patterns of lobular and ductal carcinoma vary significantly in terms of sites of distant metastases. Several studies have reported a greater tendency for lobular and mixed ductal-lobular carcinomas to metastasize to the GI tract and it usually occurs in association with spread to other sites [13, 17, 26, 33]. It has also been described that the lobular component of mixed tumours ultimately spread to the GI tract [17]; these findings were confirmed on radiological and histological examinations performed in this case.

Despite the fact that ILCs infrequently spread to the lower GI tract, it is important to differentiate them from other primary lesions as this will allow initiation of appropriate treatment and help prevent unnecessary operations. Our patient underwent an open appendicectomy nevertheless because there were some clinical and radiological signs suggestive of acute appendicitis. There were no histological features of appendicitis in the case described; however, the lumen of the appendix contained metastatic tumour deposits. Dirksen et al. [31] have suggested that metastatic adenocarcinoma to the appendix can often present as appendicitis at a late stage leading to a high incidence of perforation. Chemotherapy and endocrine therapy, often in combination, is the treatment of choice in these patients. Radiotherapy and palliative surgery (resection, GI bypasses, and debulking of metastatic disease) can be used in selected patients [13]. Some authors have also advocated closer GI follow-up for patients diagnosed with ILC [3, 34]. In a recent review article looking at patients with MBC affecting the GI tract, Ambroggi et al. [17] reported that the median overall survival is 16 months with a range of 5–41 months.

Orbital metastases from breast cancer are relatively uncommon and affect between 2% and 6% of patients with MBC [35–37]. Of those, about 5% involve the extraocular muscles only [38]. However, these metastases occur more frequently than is clinically recognised because they may affect other major organ systems where the consequences are easily detectable or they may remain small and asymptomatic. Some studies suggest that the incidence can be up to 30% in asymptomatic patients with known MBC [21, 36]. Metastatic lesions are more commonly found in the uvea than the orbit, with a ratio of approximately one to eight [21, 37]. Several studies have shown that breast cancer is the most common source of orbital metastases and accounts for 29%–50% of cases [21, 37, 39, 40]. The most common orbital symptoms that patients present with include diplopia, proptosis, pain, and decreased vision [19, 36, 37, 40]. Several case reports have also described ocular metastasis as the initial manifestation of a previously undetected primary breast carcinoma [36, 41, 42]. MRI is the diagnostic tool of choice as it can better visualize soft tissue structures and more precisely evaluate tumour metastases involving the orbital structures [20, 43]. Patients usually present during the sixth decade of life and orbital involvement occurs on average 4.5–6.5 years after the primary tumour is diagnosed [21]. The presence of orbital metastasis usually indicates extensive haematogenous spread from the primary tumour and the majority of these patients will also have simultaneous nonorbital metastases at the time of presentation [19, 21, 40, 44]. Furthermore, breast cancer has a strong tendency to localize in the orbital fat and muscle [40], as is illustrated in our case. Treatment for orbital metastases often requires a multidisciplinary approach and modalities include radiotherapy, chemotherapy, hormone therapy, surgery, and immunotherapy; however, the mainstay treatment is orbital irradiation because it can offer significant symptomatic improvement of orbital signs and symptoms [20, 40, 43]. Despite the fact that short-term visual prognosis after radiotherapy can be good, the systemic prognosis of these patients remains poor; mean survival time is reported to vary between 10 and 20 months [37, 44, 45].

## 4. Conclusion

Breast cancer has the potential to disseminate via the lymphatic or haematogenous routes and hence virtually any anatomical site can be affected with metastatic deposits [19]. Extrahepatic GI and orbital metastases from breast cancer are unusual in clinical practice. Both of these conditions can be asymptomatic or present with nonspecific symptoms, thus making their diagnosis difficult. Physicians need to recognise the different metastatic patterns of lobular and ductal carcinomas; they also need to have a high index of suspicion for metastatic disease especially in patients with a known history of breast cancer who present with new gastrointestinal complaints or orbital symptoms. For lesions in the gastrointestinal tract, it is important to distinguish a primary carcinoma from a metastatic one in order to initiate appropriate treatment in such patients and help prevent unnecessary surgical procedures. Similarly, early recognition and treatment of patients affected by ocular metastases can help preserve their vision and maximize their quality of life.
